# Special Issue “Feature Papers in Journal of Developmental Biology”

**DOI:** 10.3390/jdb10010005

**Published:** 2022-01-10

**Authors:** Robert W. Dettman

**Affiliations:** 1 Department of Pediatrics, Northwestern University, Chicago, IL 60611, USA; r-dettman@alum.northwestern.edu; 2 Perinatal Origins of Disease Program, Stanley Manne Children’s Research Institute, Ann and Robert H. Lurie Children’s Hospital, 225 E. Chicago Ave., Chicago, IL 60611, USA

Here, we have assembled five interesting manuscripts that deserve special attention. While the focus is wide, we believe that these manuscripts make a valuable contribution to the field of developmental biology.

In the first article [1], Nguyen and colleagues investigated bone remodeling and cell signaling between the following two types of bone cells: osteoblasts and osteoclasts. They report that a peptide inhibitor of osteoclast development promotes bone formation by activating ERK and promoting the expression of BMPR1a. Thus, by studying the normal process of bone remodeling, the authors have identified a peptide that shows promise as a therapy for osteoporosis.

Lewejohan and co-authors, in the second article [2], studied the effects of the disruption of the N-acetylglucosamine/N-deacetylase/N-sulfotransferase 1 (Ndst1) gene, which controls heparan sulfate (HS) biosynthesis. Heparin sulfate is a critical extracellular matrix component that is important for normal embryonic development. The difficulty in studying this gene is that there are other family members that can genetically complement its function. This is what the authors found in the central nervous system. When Ndst1 was conditionally deleted in Purkinje cells, there was no phenotype in the brain. In an effort to tease this apart, the authors also altered the function of Ndst2 in mice and, surprisingly, found only a female sterile phenotype. Thus, driving loss-of-function with the Purkinje-specific promoter Pcp2/L7, the authors found a function for heparin sulfate sulfation in female mouse reproduction. It is formally possible, but not tested, that the female sterility could be related to a CNS defect that changes the behavior of the mouse, which will likely be the author’s next step. As expected with mouse genetics, carefully planned genetic experiments often have unanticipated, but intriguing, outcomes.

In another article, Sutton et al. [3] examined gene expression in the hearts of fish that live in an extraordinary environment. These fish, Oreochromis (Alcolapia) alcalica or soda cichlids, live in the warm, hypersaline environment of Lake Natron, Tanzania. Accordingly, the question is if development in an extreme environment such as this changes the canonical gene expression pattern in heart development. The authors chose to look at the following three genes: gata4, tbx5, and mef2c. They found that, out of these three genes, gata4 had a different expression pattern, in terms of blood vessel development, than that observed in zebrafish (Danio rerio). The major accomplishment here was the rearing of the developing soda cichlid embryos in captivity and finding probes to carry out the in situ hybridizations. While just scratching the surface of their question, I hope that these authors continue to study the developmental evolution of the heart in extremophiles. Focusing our research on only a small number of model systems may, ultimately, limit our understanding of development in critical ways that we cannot anticipate.

In the fourth article [4], Roeseler and collaborators report the phenotypes associated with a hypomorphic allele of the gastrulation brain homeobox 2 gene (Gbx2). This mutant was advantageous to the authors because its less-severe phenotype allowed them to tease apart the function of the gene in hindbrain and trigeminal nerve development. They found that Gbx2 hypomorphic mice had truncated rhombomeres r(2), and that Gbx2 was required for the expression of Nrp1 in a subset of trigeminal neural crest cells. This implicated that Gbx2 plays a key role in the migration of neural crest cells into the trigeminal ganglion. This study adds to the story of how neural crest migration streams are regulated and how they contribute to hindbrain development.

Finally, Halloran et al. [5] review the role of bone morphogenetic protein-2 (BMP-2) in bone development and homeostasis. This review focuses on the potential and limitations of recombinant human BMP-2 as a therapeutic for bone disorders.

We hope that the readers enjoy these articles, not only because they make contributions to their respective fields, but also because they open us up to new ideas and new ways of thinking about development.
